# Rapid Detection of Perfluorooctanesulfonic Acid Using Surface-Enhanced Raman Spectroscopy and Deep Learning

**DOI:** 10.1021/acsomega.5c06511

**Published:** 2025-09-23

**Authors:** Aniwat Juhong, Bo Li, Yifan Liu, Cheng-You Yao, Chia-Wei Yang, A. K. M. Atique Ullah, Xuefei Huang, Mati Horprathum, Wibool Piyawattanametha, Hui Li, Zhen Qiu

**Affiliations:** † Department of Electrical and Computer Engineering, 3078 Michigan State University, East Lansing, Michigan 48824, United States; ‡ Department of Biomedical Engineering, Michigan State University, East Lansing, Michigan 48824, United States; § Department of Chemistry, Michigan State University, East Lansing, Michigan 48824, United States; ∥ Opto-Electrochemical Sensing Research Team (OEC), 54782 National Electronics and Computer Technology Center (NECTEC), Pathumtani 12120, Thailand; ⊥ Department of Plant, Soil and Microbial Sciences, Michigan State University, East Lansing, Michigan 48824, United States; # Department of Biomedical Engineering, School of Engineering, 61794 King Mongkut’s Institute of Technology Ladkrabang (KMITL), Bangkok 10520, Thailand; ¶ Institute for Quantitative Health Science and Engineering, Michigan State University, East Lansing, Michigan 48824, United States

## Abstract

Per- and polyfluoroalkyl substances (PFAS) are a large group of human-made chemicals that have been widely used in industry and consumer products. Perfluorooctanesulfonic acid (PFOS) is a ubiquitous type of PFAS, which is extremely stable chemicals that have been persistent in the environment for many years. The accumulation of PFOS in the human body can lead to various unfavorable health issues related to the immune, metabolic, and endocrine systems. The conventional PFOS detection method utilizes liquid chromatography coupled with a mass spectroscopy system that typically involves a lengthy and complex procedure. Herein, we propose to develop a low-cost and rapid test approach based on surface-enhanced Raman spectroscopy (SERS) and deep learning for PFOS detection. The gold nanoparticle SERS substrates utilized in this study can significantly enhance the Raman signal of PFOS in solution at a low concentration. PFOS detection and quantification in water using the SERS-based substrate are carried out by measuring Raman peak intensities of PFOS in solution at a range of low concentrations and comparing them to the signal of a blank SERS substrate background. The results show that the SERS substrate can achieve a detection limit as low as 0.0005 ppb. In addition, we propose a demultiplexing deep learning model, which can generate high signal-to-noise ratio (SNR) PFOS spectra from the noisy mixture of PFOS and background Raman spectra. Average cross-correlation and mean absolute error (MAE) are utilized to evaluate the similarity between the demultiplexed and denoised PFOS Raman spectra (output of deep learning) and their ground truths. The proposed model can achieve an encouraging result with high average cross-correlation and low average MAE of 0.9622 ± 0.0667 and 0.0034 ± 0.0024, respectively.

## Graphical abstract

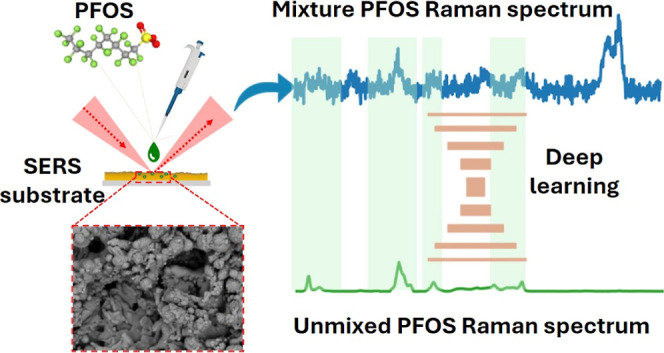

## Introduction

Surface-enhanced Raman spectroscopy (SERS) is a surface-sensitive technique that utilizes rough metal surfaces (e.g., silver or gold)^1–3^ with nano structures to enhance Raman scattering (inelastic scattering). This enhanced Raman signal is attained through localized surface plasmon resonance.^4^ This occurs in molecules located at or adjacent to nanostructured noble metal surface, resulting in significantly increasing charge transfer between the substrate and the target molecule. Indeed, SERS intensity of different target molecules greatly depends on the materials and morphology of SERS substrates, as well as the affinity between the substrates and molecules.^5–8^ Moreover, each SERS substrate has different background signals, depending on material, which should not coincide with the predominant Raman peaks for the target molecules. This is an essential factor that needs to be addressed for detecting various target molecules. Typically, the Raman signal could be enhanced to 10^10^ to 10^11^ times, which allows this technique to detect very low levels of chemicals.^9^ Thus, this powerful SERS technique is capable of detecting trace-level chemicals, and it is commonly used in various applications, such as food safety,^10,11^ biotechnology,^12,13^ surface science,^14,15^ and environmental monitoring.^16–18^

In recent years, deep learning has become a powerful tool in numerous applications, including natural language processing,^19,20^ computer vision,^21–24^ and speech recognition.^25–27^ Deep learning can unprecedentedly extract obscure features and information from complex data, and most deep learning architectures are easily adjusted to meet various requirements in applications. As a result, it has also shown a resounding success in analytical chemistry,^28–32^ specifically, in the applications of Raman spectroscopy.^33–37^

Perfluorooctanesulfonic acid (PFOS) is one of the predominantly concerned per- and polyfluoroalkyl substances (PFAS), which have been commonly utilized in a wide range of industrial processes, including stain-resistant fabrics, aqueous fire-fighting foam (AFFF), and food packaging.^38,39^ Consequently, PFOS is commonly found in human blood, wildlife, food, soil, and water in the world.^40^ Since PFOS was frequently added in AFFF, it is particularly present in soil and water at airports and other related installations.^41^ This could result in numerous severe human health issues. There are several approaches employed for PFOS detection, such as liquid chromatography/tandem mass spectrometry (LC–MS/MS), colorimetry, fluorescence spectroscopy, and potentiometry.^41–45^ In fact, these conventional methods require extraordinarily complicated and costly devices, as well as laborious preparation processes, which are not applicable for rapid detection. While SERS is an effective and affordable approach for the rapid PFOS detection,^46–48^ the computational analysis would be required to distinguish the identities of PFOS spectra from the mixture spectra. In practical implementation, PFOS spectra tend to be scrambled with other spectra, particularly in complicated sample matrices. The comparison between the proposed method and other conventional methods for PFOS detection is also shown in Table 1.

**Table 1:** Comparative Table Summarizing Cost per Sample, Analysis Time/Complexity, and Limit of Detection (LOD) of Representative Conventional Methods vs. the Proposed Method

method	cost per sample (unit: USD)	analysis time/complexity	LOD
chromatographic methods	high (>200)	could be up to several hours, depending on users’ experience/high complexity	0.0016 ppb^49,50^ 7–40 ppb^51^
total oxidizable (TOP) assays	high (>300)	1–6 hours/high complexity	0.0005–0.0079 ppb^49,52–54^
electrochemical methods	low (<50)	rapid test (less than 30 minutes)/low complexity	0.0017 ppb^55,56^
our method	low (<10)	rapid test (less than 30 minutes) /low complexity	0.0005 ppb

In this work, we report an approach based on SERS, integrated with the demultiplexing deep learning model, to detect PFOS in water, which is a predominant PFOS-contaminated source. The SERS substrates utilized in this research are custom-made gold nanoparticle substrates employed for the serial dilution of PFOS solution. The detection limit observed from this serial dilution experiment can achieve the detection limit as low as 0.0005 ppb. To verify the ability of the SERS substrate to detect PFOS solution, we compared Raman spectra of PFOS on the SERS substrate to PFOS powder and the blank SERS substrate (background signal). The result shows that the Raman signal at 1,044 cm^–1^ of the PFOS solution at low concentrations (from 5 μg/L to 0.00005 μg/L) on the SERS substrate is the same as that of the PFOS powder and not covered by the background signal. This compelling result allows us to develop reliable detection and quantification of PFOS at a relatively low concentration using the SERS substrate. Furthermore, a deep learning-based approach enables the unmixing of PFOS spectra from the background signal, facilitating effortless analysis of the PFOS spectra. The proposed deep learning model can effectively unmix and generate high SNR PFOS spectra with an average cross-correlation and mean absolute error between the deep learning outputs and ground truths of 0.9622 ± 0.0667 and 0.0034 ± 0.0024, respectively.

## Materials and Methods

### Sample Preparation and Raman Measurement

PFOS was purchased from TCI America (Portland, OR, USA) with a purity of 98.0%. Since PFOS has water solubility of 680 mg/L,^57^ we prepared 50 mL of PFOS solution with a high concentration of 500 mg/L, which was then diluted to 500 μg/L (ppb) using ethanol as the solvent. The 500 ppb PFOS stock solution was further diluted with deionized (DI) water to 5, 0.5, 0.05, 0.005, 0.0005, and 0.00005 ppb. Using DI water to dilute the solution can substantially reduce the Raman background signal from ethanol, and less than 1% of ethanol in water had negligible effects on background signals. Each PFOS solution (15 μL) was dropped on the SERS substrate (ONSPECT-Lite, National Electronics and Computer Technology (NECTEC), Bangkok, Thailand). The solution on the substrate was then dried out at room temperature, followed by promptly acquiring Raman spectra using a Renishaw inVia Raman spectrometer, which is connected to a Leica microscope (Leica DMLM, Leica Microsystems, Buffalo Grove, IL, USA). A 785 nm near-infrared (near-IR) laser with a power of 15 mW, a 20× NA = 0.45 objective lens (Leica Microscope), and a 3,000 ms exposure time with an average number of 10 accumulations were used for the data acquisition of each scanning position. A general overview of this work is also demonstrated in Figure 1.

**Figure 1:**
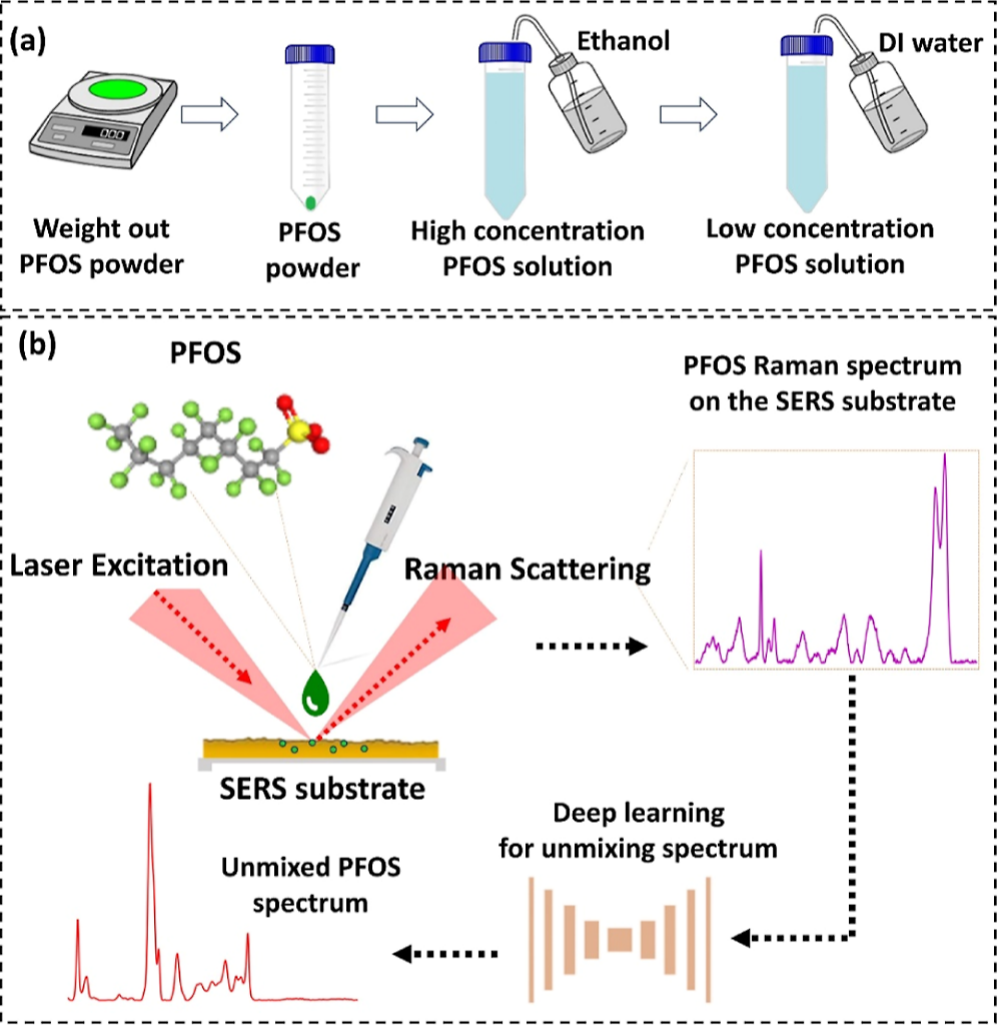
Schematic of the proposed method based on surface-enhanced Raman spectroscopy (SERS) and deep learning for PFOS detection. (a) PFOS solution preparation, PFOS powder is first weighted out and ethanol is used to dissolve the PFOS powder to obtain the PFOS solution with a high concentration. DI water is then added to dilute the PFOS solution. (b) Overview of the proposed method is that the PFOS solution is dropped on the SERS substrate, followed by acquiring the enhanced Raman spectra of PFOS. Deep learning is utilized to unmix the Raman spectrum of PFOS from the mixture spectrum (PFOS and background).

The gold nanoparticle SERS substrate used in this study is a cost-effective substrate (less than 10 USD per sample) widely used for numerous applications, particularly rapid detection in trace chemical and biological analysis.^58–61^ The substrate was fabricated by the Opto-Electrochemical Sensing Research team at National Electronics and Computer Technology Center (NECTEC), Bangkok, Thailand. In short, a laser-making machine is employed to create nanoto-microscaled roughness on the surface of a metal sheet. Noble metal nanoparticles with an average size of 59 ± 17 nm are then deposited on the roughened metal sheet, creating a 3D-strcutured SERS substrate with noble metal nanoparticles adhered to the roughened surface. The photograph and scanning electron microscope (SEM) images of the SERS substrate are shown in Figure 2. More details of the fabrication process and characterization of the SERS chip can be found in a previous publication by the NECTEC research group.^62^

**Figure 2:**
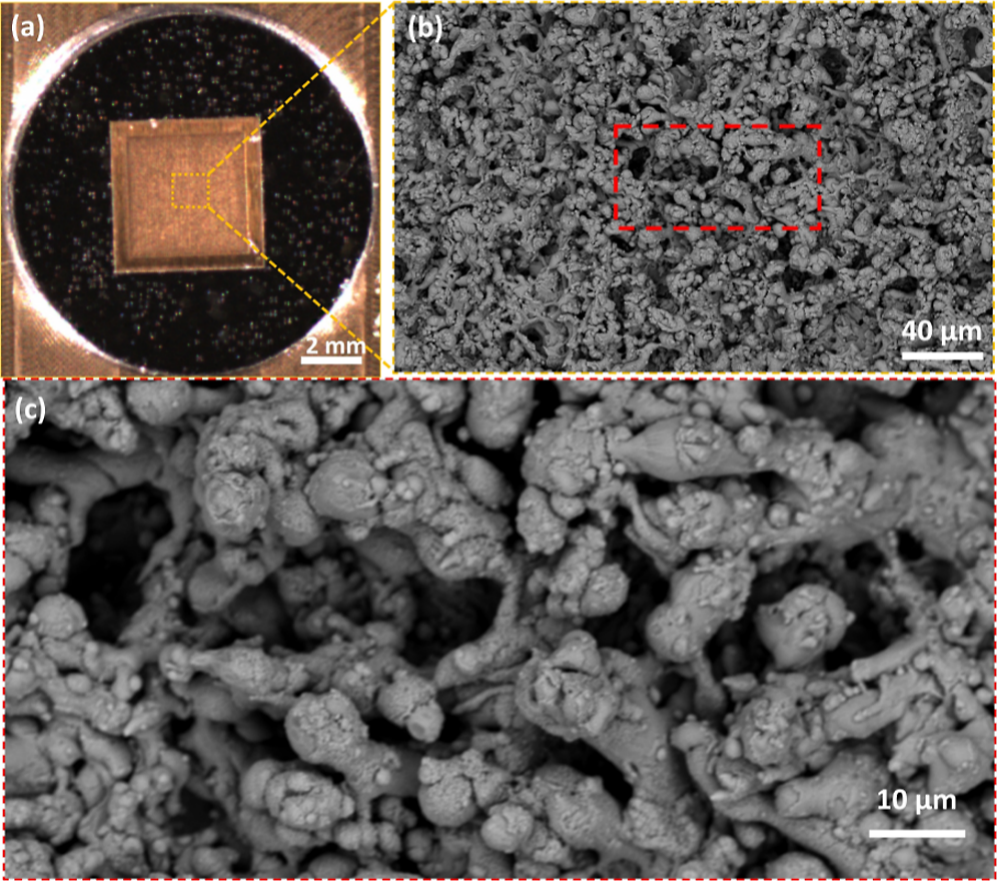
Gold nanoparticle SERS substrate used in this work (ONSPECT-Lite, NECTEC, Bangkok, Thailand). (a) Photograph of the SERS substrate. (b) Scanning electron microscope (SEM) image of the SERS substrate. (c) The enlarged SEM image in the red-dashed box in (b).

### Deep Learning Model

The encoder-decoder, together with the skip connections, also known as the UNet architecture, are employed to denoise and unmix the low SNR mixture spectra (input of the deep learning model), as shown in Figure 3a. The encoder is first employed for downsampling the input tensor. Each encoder block consists of ResNet convolutional and Max-Pooling blocks. The ResNet convolutional block is applied to capture and extract latent features of an input tensor. Its architecture is assembled from convolutional blocks with a kernel size of 3, a stride and padding of 1, a PReLU activation function, and a skip connection. Max-Pooling reduces the tensor size by half for each encoder block. The decoder block is composed of convolutional transpose for upsampling the tensor, followed by the ResNet convolutional blocks. The last decoder block applies the Tanh function as its activation function. The output of this function varies from −1 to 1. The advantage of using this function is that it avoids exploding and vanishing output values. Consequently, the input is normalized to align with the output range of −1 to 1. Skip connections are also added between each encoder and decoder, allowing the gradient to easily backpropagate in order to optimally update the weights. Furthermore, the Transformer encoder is used to extract the features from the same input (the low SNR mixture Raman spectrum). The output of the Transformer is then multiplied with the output of the bottleneck layer of UNet, as shown in Figure 3a. The Transformer encoder begins with patch embedding, using a learnable linear projection to map the vectorized input patches ( $x_{p}^{n}$ ) into a latent dimensional embedding space. Position embeddings are then added to the patch embeddings to maintain positional information, as shown in eq 1.

1 $$Z_{0}=\left[x_{p}^{1}E;x_{p}^{2}E;...;x_{p}^{n}E\right]+E_{pos}$$

where *E* is the patch embedding projection, *E*_pos_ denotes the position embedding. The Transformer encoder is composed of L layers of Multi-head Self-attention (MSA) and Multilayer Perceptron (MLP) blocks shown in eqs 2 and 3, the output of the γ-th layer can be expressed as follows:

2 $$Z_{\gamma }^{\prime }=MSA\left(LN\left(Z_{\gamma -1}\right)\right)+Z_{\gamma -1}$$

3 $$Z_{\gamma }=MLP\left(\mathrm{LN}\left(Z_{\gamma }^{\prime }\right)\right)+Z_{\gamma }^{\prime }$$

where LN(·) is the layer normalization operator and Z_γ_ is the encoded spectral representation. The architecture of a Transformer layer is shown in the bottom right of Figure 3a. Herein, the size of each input tensor (mixture Raman spectra) is 1 × 1 × 896 and it is fed to the encoder blocks (E1, E2, E3, and E4), followed by the bottleneck block (B), and the decoder blocks (D1, D2, D3, and D4), respectively.

**Figure 3:**
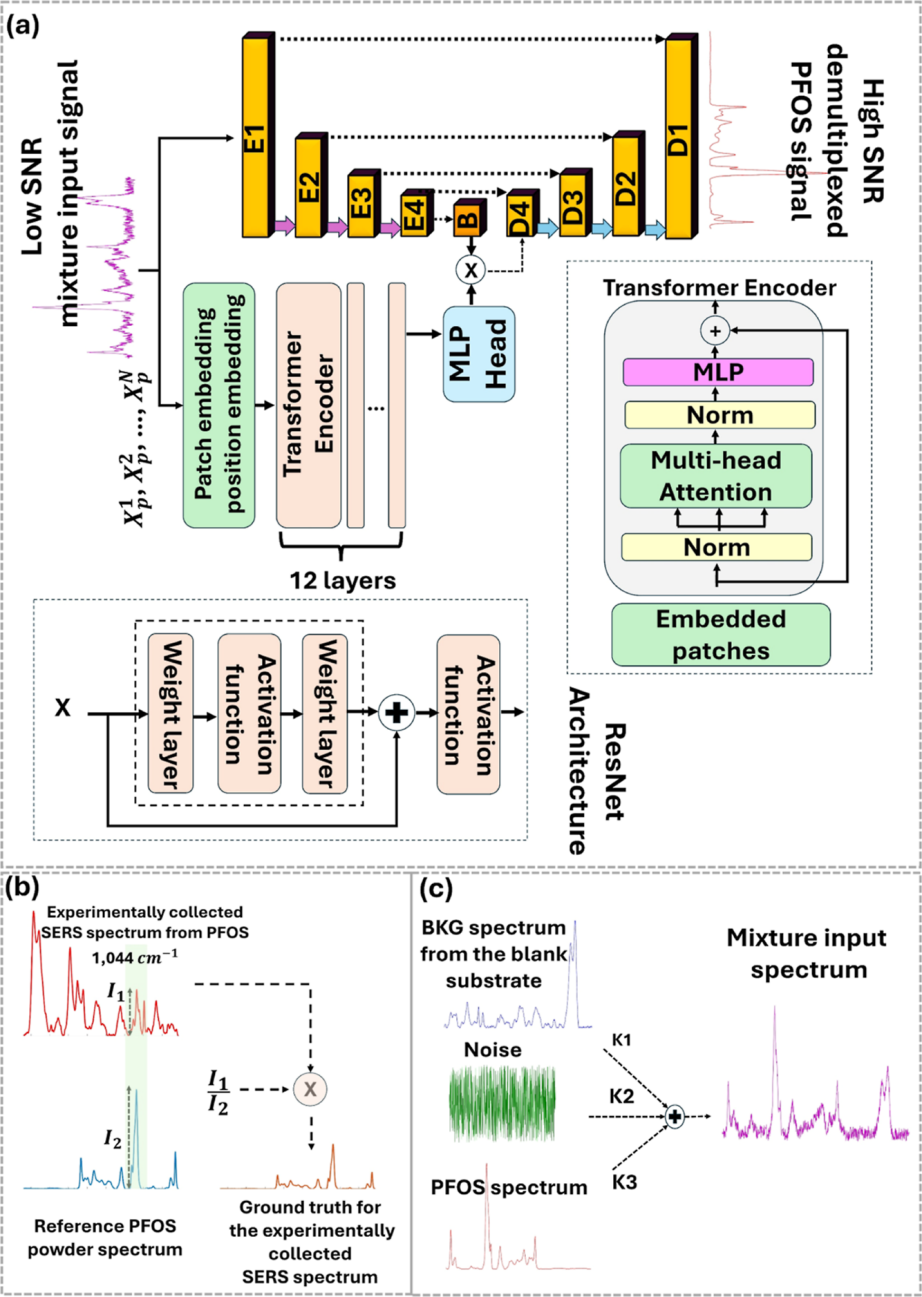
Proposed deep learning model and data set preparation. (a) Transformer UNet encoder-decoder for denoising and demultiplexing the Raman spectrum. (b,c) Schematics of the data set preparation for experimentally collected data and simulated data, respectively.

### Data Set Preparation

To prepare the data set for training the proposed deep learning model, the mixture of noisy input spectra was simulated using the pure Raman spectra of the SERS substrate background (a blank substrate), PFOS powder (reference of the PFOS spectrum), and a Gaussian noise signal. Each spectrum was multiplied by a random constant, followed by combining them together, as shown in eq 4. All the constants (K1–K3) were random without a single repeat. In total, we simulated 1,080 spectra, 120 spectra, and 30 spectra for training, validation, and testing data sets, respectively.

4 Simulatedmixturespectrum=(K1)(BKG)+(K2)(PFOS)+(K3)(GN)

where K1 and K2 are random constants in the range of 0.5 to 5. K3 is derived from the highest intensity of the mixture signal between (K1)­(BKG) and (K2)­(PFOS) multiplied by a random constant in the range of 0.01 to 1. BKG, PFOS, and GN represent the Raman spectra of the background substrate, PFOS powder, and Gaussian noise, respectively. Accordingly, the ground truth of each simulated spectrum is determined as (K2)­(PFOS).

Apart from the simulated data set, the experimentally acquired surface-enhanced Raman spectra of PFOS (acquired from the serial dilution experiment) were also employed for training and testing deep learning models with the three following steps for their corresponding ground truth preparation:

Step 1: Remove the baseline of Raman spectra of PFOS on the SERS substrate (obtained from the experiment) and PFOS powder/reference. Therefore, their baselines are set to be at an analogous level.

Step 2: Determine the spectral intensity at the wavenumber of 1,044 cm^–1^ for the PFOS on SERS and the PFOS powder. This wavenumber is the predominant peak of the PFOS reference signal and does not coincide with the background of the SERS substrate. The intensity ratio at 1,044 cm^–1^ between the PFOS on SERS over the PFOS powder is then computed for employing in the next step.

Step 3: Multiply the PFOS powder signal by the ratio derived from Step 2 to estimate the ground truth of the PFOS on SERS signal.

In total, there are 630 spectra of the experimentally collected data, acquired from PFOS solutions with 5 different concentrations (5, 0.5, 0.05, 0.005, and 0.0005 ppb) on the SERS substrates. However, the size of data is somewhat small for training a deep learning model.

Thus, the augmentation is applied to the training data by multiplying a random factor and adding random Gaussian noise as shown in eq 5, where C1 and C2 were random factors without a single repeat. This augmentation was only applied to 600 spectra to double the size of the data to 1,200 spectra, but 30 other spectra are reserved for the testing data set (the total original size of all experimentally collected data comprises 630 spectra). The 1,200 spectra are then divided into the 1,080 and 120 spectra for training and validation data sets, respectively.

5 Actualmixturespectrum=(C1)(PFOSonSERS)+(C2)(noise)

The overview schematics of data preparation are also demonstrated in Figure 3b,c. In essence, the simulated and actual measurement data sets are merged for training, validation, and testing the models. In summary, there are 2,160, 240, and 60 spectra for training, validation, and testing data sets, derived equally from both the simulated and experimentally collected data sets. It is important to note that the size of simulated data set should not be significantly larger or smaller than the actual measurement data set in order to circumvent the overfitting problem.

### Training Implementation

The proposed model was trained on a personal computer with an Intel Core i7-9750U CPU, 64 GB RAM, and an NVIDIA RTX 3090 graphics card, using Pytorch version 2.0.1 library with the following hyper parameters: learning rate of 0.0001, number of epochs of 50, and batch size of 8. The Adam optimizer and mean absolute error (MAE) were used for training the model.

6 $$MAE=\frac{\sum_{i=1}^{n}\left|y_{i}-x_{i}\right|}{n}$$

where *y*_*i*_ is the ground truth, *x*_*i*_ is the predicted output, and n is the total number of spectra.

## Results and Discussion

### Serial Dilution of PFOS Solution

PFOS has a molecular structure consisting of a hydrophobic perfluorooctane tail in conjugation with a sulfonate headgroup, as shown in Figure 4a. Typically, the vibrations of each bond in PFOS demonstrate distinct Raman wavenumbers with their intensity correlating to the concentration of PFOS. In this experiment, PFOS solutions at six concentrations of 5, 0.5, 0.05, 0.005, 0.0005, and 0.00005 ppb were dropped on the six different SERS substrates. After that, 36 positions of each substrate or concentration were scanned using a Raman spectrometer to acquire the 36 Raman spectra, followed by averaging them. In addition, the average spectrum of PFOS powder was also acquired to compare with the average SERS of the PFOS solutions for evaluating their Raman wavenumbers’ similarity, as illustrated in Figure 4a.

**Figure 4:**
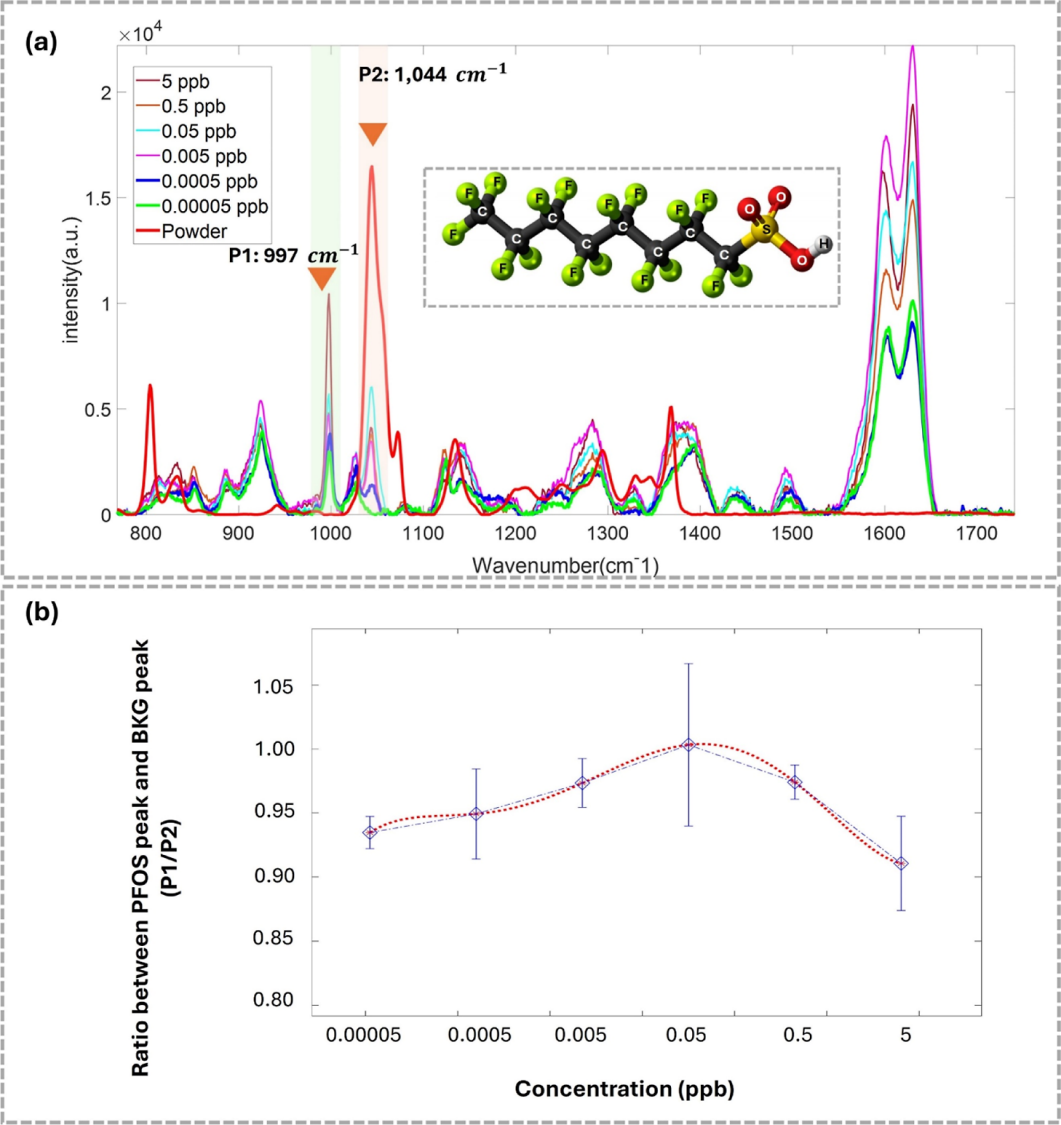
PFOS detection using SERS and its calibration curve result. (a) The average Raman spectra of PFOS solution at different concentrations (solvent: DI water). (b) The calibration curve of PFOS solution (*x*-axis is the PFOS concentration and *y*-axis is the ratio between P1 (997 cm^–1^) to P2 (1,044 cm^–1^)) of the Raman spectra in (a).

According to the serial dilution result, two Raman peaks at 997 cm^–1^ (P1, the assignment of C–C^63,64^ ) and 1,044 cm^–1^ (P2, the assignment of S–O_3_ ^47^) of the average SERS PFOS spectra correspond closely to the Raman spectrum of the PFOS powder. Furthermore, the intensities of these two peaks show considerable change when the PFOS concentrations also differ. It is crucial to point out that the P2 peak is the same peak observable in both PFOS powder and PFOS SERS spectra, where the P2 peak can be obviously observed from PFOS solution with concentration from 5 to 0.0005 ppb. The lowest concentration that P2 can still be noticeable is 0.0005 ppb (blue plot in Figure 4a), whereas the P2 peak completely vanishes at 0.00005 ppb (green plot in Figure 4a).

### Background and PFOS Raman Spectra

To alleviate the spectral variability, the relative ratio between P1 and P2 was utilized for plotting the calibration curve as shown in Figure 4b. Typically, the intrinsic Raman spectra intensity of a lower concentration solution should be weaker than the intensity of a higher concentration solution. However, the SERS Raman intensity greatly depends on the electromagnetic and chemical enhancement effects between the nanosurface and the target molecule to create hotspots. Increasing concentration could lead to large particle sizes, resulting in poor bonding with the nanoparticle of the substrate and lowering the signal enhancement. In addition, unbound target molecules could potentially block the enhanced signal. In our experiment, the PFOS solution at a higher concentration (5 and 0.5 ppb) showed progressively decreased intensity of Raman spectra, while the lower concentration solution at 0.05 ppb showed a stronger intensity. To validate that the Raman spectra of PFOS on the SERS substrate are genuinely obtained from PFOS molecules enhanced by the substrate, the background signal (BKG) of the substrate was also acquired, followed by comparison with the PFOS powder and the PFOS SERS spectra. Since the PFOS SERS at a concentration of 0.05 ppb shows the highest intensity of the P2 peak compared to other concentrations, it was employed for this comparison. As previously mentioned, the Raman peak at 1,044 cm^–1^ (P2) only appears in the PFOS powder and PFOS SERS spectra and it does not appear in the background spectra of the blank SERS substrate, which is substantiated by the result, as shown in Figure 5 below. However, only one nonoverlapping peak and some overlapping peaks are somewhat challenging to comprehensively analyze the result. Therefore, as discussed in the next section, we propose a deep learning model to demultiplex the mixture spectra between PFOS and background spectra.

**Figure 5:**
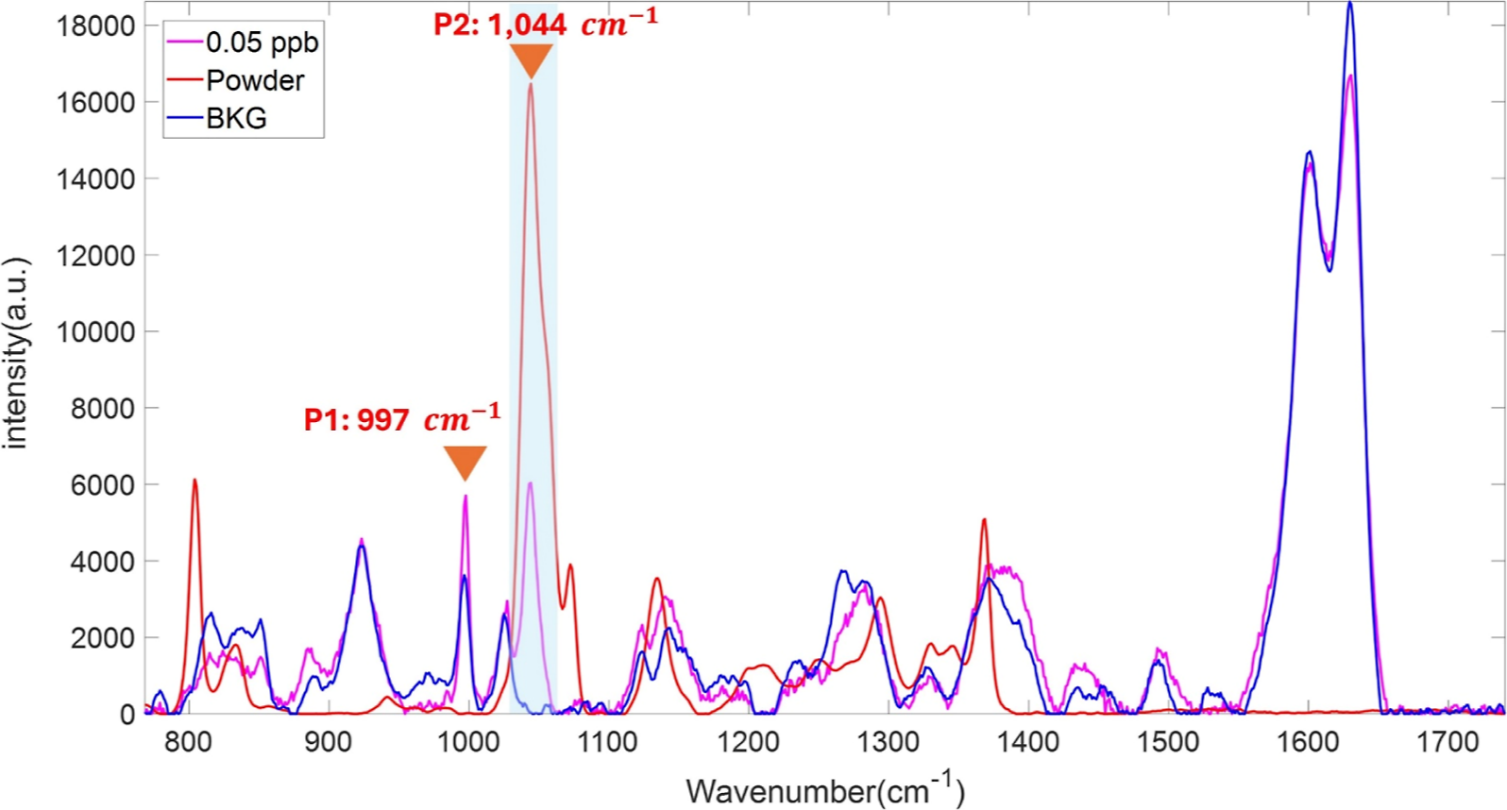
Comparison of PFOS SERS (magenta spectrum) acquired from the PFOS solution with a concentration 0.05 ppb, PFOS powder (red spectrum), and a blank SERS substrate (blue spectrum).

### Demultiplexing Deep Learning for PFOS Raman Spectra

In practical implementation, Raman spectra of an unpurified and inhomogeneous sample typically originate from mixture spectra of several compounds, especially SERS, which includes a strong background signal of the substrate itself. To accurately analyze the Raman data, we need an algorithm to extract the signal of the compound that we desire to detect. Therefore, we propose a deep learning model to demultiplex the PFOS Raman spectra from the mixture spectra of PFOS and SERS background. Apart from demultiplexing, the proposed deep learning model can also enhance the signal-to-noise ratio (SNR) of the demultiplexed output. In short, the proposed deep learning model can simultaneously perform demultiplexing and SNR enhancement to retrieve the high SNR and unmixed PFOS spectra. To evaluate the model’s performance, the testing data set (unseen data and not used for training), cross-correlation,^65^ and MAE were utilized. Cross-correlation is a function commonly used to evaluate the similarities of two signals, which can be defined as the following equation:

7 $$R=\frac{\sum \left(x_{i}-\mathrm{x̅}\right)\left(y_{i}-\mathrm{y̅}\right)}{\sqrt{\sum {\left((x_{i}-\mathrm{x̅})\right)}^{2}\sum {\left((y_{i}-\mathrm{y̅})\right)}^{2}}}$$

where *R* is the correlation coefficient, *x*_*i*_ is the value of the predicted output, x̅ is the mean of the predicted output, *y*_*i*_ is the ground truth, y̅ is the mean of the ground truth, and i is the number of sampling points of the signal (0,1,2,3, ..., *n* – 1). Figure 6 shows the representative results of the testing data set. The mixture input spectra (dark blue spectra) from both simulated data and experimentally collected data were employed for testing three different deep learning models: UNet, ResNet UNet, and Trans UNet, with their outputs illustrated as purple, red, and green spectra, respectively. The generated Raman spectra were compared to the corresponding ground truth spectra (orange spectra) by using two average metrics, cross-correlation and MAE, as presented in Table 2. Overall, all the models can exceptionally perform on the demultiplexing and SNR enhancement. The state-of-the-art Trans UNet model outperforms the other models.

**Figure 6:**
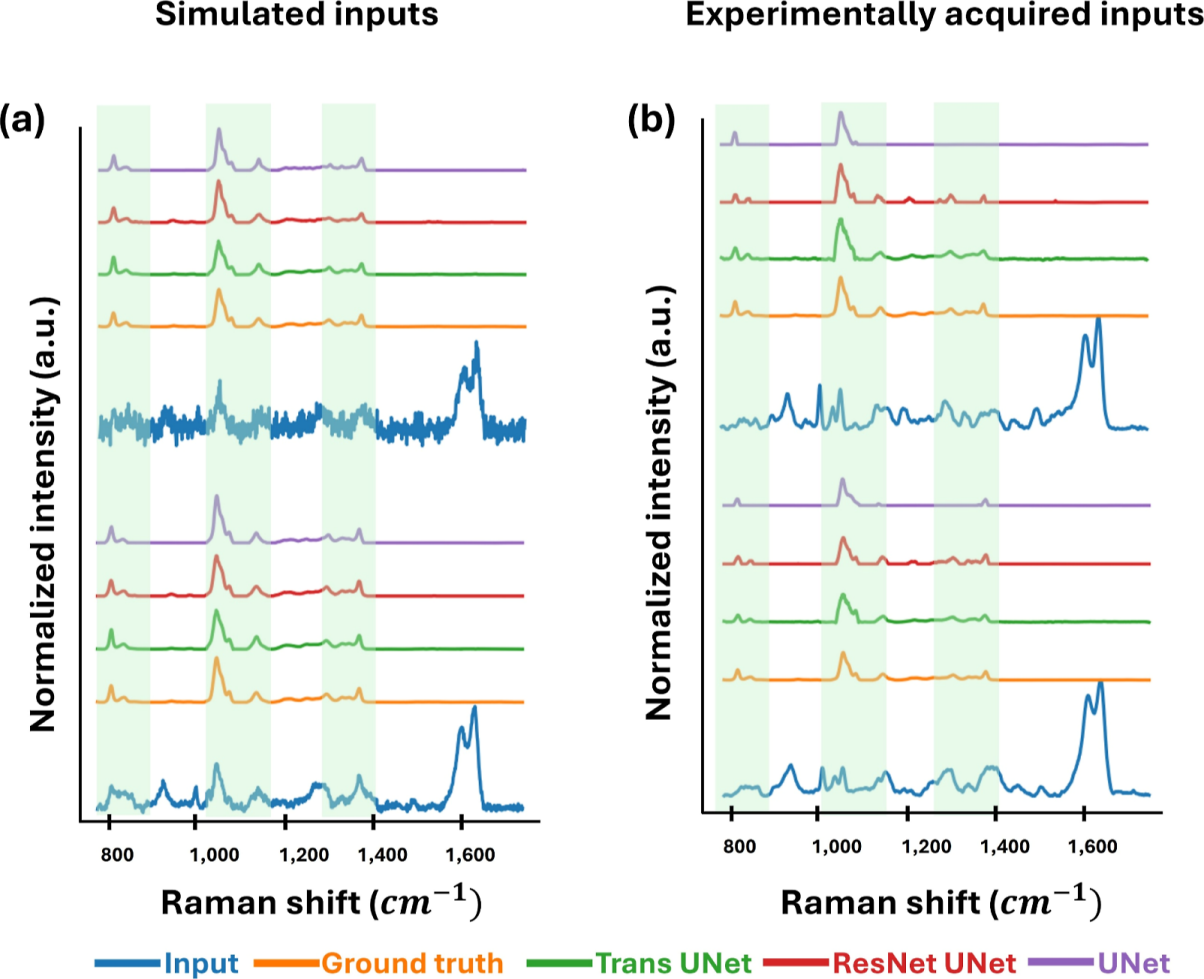
Representative demultiplexing results of three deep learning models: Trans UNet, ResNet UNet, and UNet. (a,b) The results from simulated data and experimentally collected data, respectively. From the bottom to the top, mixture spectrum of PFOS and SERS (input), ground truth PFOS reference spectrum, deep learning results from the three deep learning models. The common peaks between the PFOS signals and the mixture spectrum are highlighted in green.

**Table 2:** Performance Comparison of Three Different Deep Learning Models for PFOS Raman Demultiplexing (Average ± Standard Deviation)

	UNet	ResNet UNet	Trans UNet
average cross-correlation	0.8582 ± 0.2667	0.9043 ± 0.2148	0.9622 ± 0.0667
average MAE	0.0046 ± 0.0035	0.0036 ± 0.0030	0.0034 ± 0.0024

## Conclusion

In this work, we present an approach based on SERS together with deep learning for PFOS detection, showing promising results. The SERS substrate can significantly enhance the Raman spectra of PFOS in solution and detect the trace amount of PFOS solution down to 0.0005 ppb. In addition, the proposed deep learning model, which is employed for demultiplexing and SNR enhancement of low-SNR mixture spectra of PFOS and background spectra, shows promising evaluation result, where the average cross-correlation and MAE are 0.9622 ± 0.0667 and 0.0034 ± 0.0024, respectively. This could ease the difficulty of using the SERS technique to detect PFOS, as we can easily obtain the unmixed PFOS spectra from the mixture Raman spectra. With these promising results, we anticipate that the proposed method could be an alternative and practical method for PFOS detection. In the future, we will explore more feasibility of employing the proposed method to detect the PFOS in the natural environment, especially water, soil, and human blood.

## Data Availability

The SERS data and code are publicly available at https://github.com/AniwatJuhongNACK/Deep-learning-for-PFOS-Raman-spectra.
